# Suspected Hepatotoxicity With a Supercritical Carbon Dioxide Extract of *Artemisia annua* in Grapeseed Oil Used in New Zealand

**DOI:** 10.3389/fphar.2019.01448

**Published:** 2019-12-20

**Authors:** Ruth L. Savage, Geraldine R. Hill, Joanne Barnes, Susan H. Kenyon, Michael V. Tatley

**Affiliations:** ^1^New Zealand Pharmacovigilance Centre, Dunedin School of Medicine, University of Otago, Dunedin, New Zealand; ^2^Department of General Practice, University of Otago, Christchurch, New Zealand; ^3^Medsafe, Ministry of Health, Wellington, New Zealand; ^4^School of Pharmacy, Faculty of Medical and Health Sciences, The University of Auckland, Auckland, New Zealand

**Keywords:** *Artemisia annua*, herb-induced liver injury (HILI), herbal medicines, pharmacovigilance, adverse reaction reporting

## Abstract

A case series of hepatotoxicity associated with an extract of *Artemisia annua* L. was identified through the New Zealand spontaneous adverse drug reaction reporting system. *A. annua* extract, produced using a supercritical carbon dioxide extraction method and formulated with grapeseed oil, has been marketed in New Zealand as a natural product for joint health. As of 31 January 2019, the New Zealand Pharmacovigilance Centre had received 29 reports of hepatic adverse reactions occurring in patients taking *A. annua* extract in grapeseed oil. The case reports were assessed for patient and adverse reaction characteristics, patterns of *A. annua* extract use and causality (based on the WHO-UMC system for standardized case causality assessment). Patients were aged 47 to 93 years (median 67). Time to onset of hepatotoxicity from starting *A. annua* extract was 7 days to approximately 12 months in the 23 reports with this information. Nineteen of these reports indicated onset within 12 weeks. *A. annua* extract was the sole suspect medicine in 27 reports. A few patients had possible predisposing conditions. Twenty-seven patients were reported to have recovered or improved on stopping *A. annua* extract. Nine patients required hospital admission. The pattern of hepatic injury varied. Jaundice, often with pruritus and dark urine, was experienced by 16 patients. There was considerable consistency across case reports from various reporters. We assessed the case reports as a series using the Bradford Hill guidelines for causal inference and concluded that there was a safety signal of a causal association between the *A. annua* extract and hepatotoxicity sufficient to be communicated and investigated further.

## Introduction

Use of the herb *Artemisia annua* L. in traditional Chinese medicine (TCM) can be traced back two millennia (Hsu, 2006; Graziose et al., 2010; Tu, 2011). In the fourth century CE, Ge Hong recommended *qinghao* (*A. annua*, sweet wormwood, family *Asteraceae*) for treating intermittent fever. Reference to the use of *A. annua* herb as an anti-inflammatory can be found in TCM texts from at least 200 CE (Hsu, 2006; Graziose et al., 2010).

Interest in the herb increased in the late 1960s, when Chinese scientists demonstrated efficacy against the malaria parasite *Plasmodium falciparum*. The active constituent *qinghaosu* (essence of *qinghao*) was isolated in 1972, and later named artemisinin (Tu, 2011). Artemisinin-based combination therapy is now the recommended treatment for uncomplicated *P. falciparum* infections (World Health Organization, 2015). In recent years, *A. annua* herb has also gained attention as a potential remedy for inflammatory conditions (Wang et al., 2011; Hunt et al., 2015; Shi et al., 2015; Wang et al., 2019).

In New Zealand, products containing a supercritical carbon dioxide (SC-CO_2_) extract of dried *A. annua* plant material (whether whole plant or plant parts not labeled) in grapeseed oil have been promoted for supporting joint health and mobility. Up to 31 January 2019, the New Zealand Pharmacovigilance Centre (NZPhvC) received 29 reports of hepatic disorders suspected to be associated with *A. annua* extract. Two products were mentioned in adverse reaction reports: Arthrem^®^ (Promisia, 2019) and GO-Arthri^®^ (GO Healthy, 2019). Arthrem^®^ is available as 150 mg capsules for twice-daily dosing and GO-Arthri^®^ (no longer marketed) was available as 300 mg capsules for once-daily dosing. Both products have been sold in bottles of 60 capsules.

The accumulating case reports suggested an emerging safety signal. In February 2018, Medsafe, the New Zealand medicines regulator, issued a safety communication regarding 14 reports of hepatotoxicity in patients taking *A. annua* extract (Medsafe, 2018a). Following the communication, a further 11 reports were received, including one report of hepatic cirrhosis, prompting a further communication in November 2018 (Medsafe, 2018b).

This article describes the characteristics of the case series and assesses the likelihood of a causal association.

## Methods

We identified all case reports in the NZPhvC database that included *A. annua* extract as a suspect medicine and a hepatobiliary system organ class (SOC) adverse reaction term (WHO Adverse Reaction Terminology). Where important information was missing from a report, we requested further details from the reporter.

Patterns of hepatic injury were determined from the reported initial liver function tests by calculating the *R* value. The *R* value is defined as the ratio of alanine aminotransferase (ALT) to alkaline phosphatase (ALP) values expressed as multiples of the upper limit of normal (ULN). Normal ranges used were those quoted in the reports or otherwise those used by the New Zealand Southern Community Laboratory. https://www.wellingtonscl.co.nz/PathologyTestGuide/TestGuide/. 

Three authors (RS, GH, JB) independently assessed the likelihood of a causal relationship between *A. annua* extract and the hepatic events for each case using the WHO-UMC System for Standardized Case Causality Assessment (Uppsala Monitoring Centre, 2018). This system grades reports as “certain," “probable,” “possible,” “unlikely” or “unassessable.” For a causality grading of “certain” or “probable,” the report had to include information indicating that other possible causes of hepatitis or hepatic injury had been excluded. In particular, the report had to include information that viral serology and, unless the pattern of liver injury was clearly hepatocellular, liver imaging had been performed and did not suggest an alternative cause. A “possible” grading was applied where there was less certainty that other causes could be excluded, including reports that were missing information on appropriate investigations or the outcome of the adverse event. We endeavored to reach a consensus decision for each report through discussion. Where consensus was not reached, a further expert opinion (MT) was sought.

## Case Series

As at 31 January 2019, the NZPhvC database contained 29 case reports of hepatobiliary SOC adverse reactions in which *A. annua* extract was reported as a suspect medicine. The reports are summarized in **Table 1**.

**Table 1:** Summary characteristics of reports of hepatic adverse reactions associated with *Artemisia annua* extract (n = 29) in the New Zealand Pharmacovigilance Centre Database.

Patient Characteristics		
M:F	10:19	
Age range, mean and median (y)	47–93, 67.8, 67	
Indications (n **=** 21)	Joint pain/arthralgia	[12]
	Arthritis/osteoarthritis	[7]
	Joint health	[2]
Relevant co-morbidities (n **=** 3)	Chronic hepatitis B with normal hepatic function	[1]
	Recent Epstein-Barr virus infection	[1]
	Small bowel obstruction	[1]
**Product Characteristics**		
*A. annua* extract sole suspect		[27]
*(sole medicine in 13/27)*		
Co-suspects	Melatonin, turmeric	[2]
*A. annua* extract dose (150 mg bd or 300 mg daily recommended) (n **=** 18)	150 mg daily	[7]
	150 mg bd	[10]
	≥150 mg bd	[1]
*A. annua* extract duration to reaction onset range, mean, median (days) (n **=** 13)	7–210, 55.3, 30	
**Adverse Reaction Characteristics**		
Clinical presentation	Hepatic enzymes increased only	[8]
	Hepatic enzymes increased or hepatitis with signs/symptoms:	[20]
	*Jaundice +/- dark urine, at or soon after presentation* *and/or*	*[16]*
	*Abdominal pain, vomiting, nausea, fever, headache, anorexia, malaise, fatigue or lethargy singly or in combination.*	*[13]*
	Hepatic cirrhosis	[1]
Seriousness	Hospitalisation	[8]
	Medically significant	[14]
Severity (peak serum concentrations across reports)	Bilirubin 608 µmol/L, alanine transferase 3311 U/L, aspartate transaminase 1976 IU/L, alkaline phosphatase 512 U/L. One patient had hypoalbuminemia.	
Hepatic injury pattern (cirrhosis report excluded) (n = 20)	Hepatocellular	[7]
	Cholestatic	[3]
	Mixed	[9]
	Mixed - > cholestatic (biopsy hepatocellular)	[1]
**Outcome**		
Dechallenge	Recovered or recovering	[27]
	Unknown	[2]

Reports were submitted by consumers (n=3), general practitioners (n=13) hospital doctors (n=3), specialist physicians (n = 3) and pharmacists (n=2). Five cases were reported more than once from different sources. The reports were received from throughout New Zealand. Further information was requested for 25 reports and received in 21 instances.

### Patient Characteristics

The 29 patients were middle-aged or older, and mostly female (n = 19). *A. annua* extract was predominantly taken for joint pain or arthritis.

Liver function prior to starting *A. annua* extract was reported for ten patients. For nine patients normal liver function was reported ranging from six months to seven years prior to starting *A. annua* extract. Three of these patients had two or more measurements. One patient had a mildly elevated serum ALT prior to starting *A. annua* extract.

### *A. annua* Extract Exposure

All 29 patients were reported to have taken the Arthrem^®^ branded product. Two patients had changed from Arthrem^®^ to Go-Arthri^®^ 6 months and 10 days, respectively, prior to onset of symptoms of hepatotoxicity. *A. annua* extract was the sole suspect medicine in 27 reports, and in 13 of these cases no concomitant medicines were reported.

Dose was recorded in 18 reports. One patient was reported to have exceeded the recommended daily dose of 300 mg. Information on the time to onset (TTO) of hepatic dysfunction from commencing use of *A. annua* extract was provided in 23 reports. In these reports, TTO ranged from 7 days to approximately 12 months, with the majority (19 of 23) indicating onset within 12 weeks. In 10 reports the TTO was calculated approximately as the start date of *A. annua* was not recorded precisely. For 13 patients with precise information TTO ranged from 7 to 210 days, (median 30). In three cases, hepatotoxicity was diagnosed 3 days, two weeks and three months, respectively, after the patient had stopped taking *A. annua* extract.

### Clinical Presentations of the Suspected Hepatic Reactions

Twenty three reports described “hepatic enzymes increased”/​“hepatic function abnormal.” Five patients experienced “hepatitis,” and one patient experienced “hepatic cirrhosis.” “Jaundice,” often presenting early, was also reported for 16 patients, including seven with “pruritus.” Two reports were suggestive of impaired hepatic synthetic function: one patient had hypoalbuminemia and another experienced purpura and hematuria.

### Patterns of Hepatic Injury

Information on the pattern of liver injury was available in 20 reports, either specifically reported or determined from information on liver function provided in the reports. As shown in **Table 1**, hepatocellular, cholestatic, and mixed patterns were all reported. For the remaining nine patients, one was reported to have hepatic cirrhosis, two had ALT and ALP values less than twice the ULN suggesting liver adaptation rather than injury, and six reports contained insufficient information to determine the pattern of liver injury.

### Seriousness and Severity

None of the reports described death as an outcome. However, nine patients required hospital admission.

In 16 reports, the hepatic adverse effects were described by the reporter as “severe.” Liver function test results were available in 21 reports. Peak concentrations of serum bilirubin, ALP, and ALT were reported for 18, 19, and 21 patients, respectively. Serum bilirubin ranged from 5 to 608 µg/L, (mean 115.3); ALP 73 to 594 IU/L (mean 307.5); and ALT 37 to 3,311 IU/L (mean 517.6).

### Response to *A. annua* Dechallenge

Twenty-seven reports indicated that the patient had stopped taking *A. annua* extract and had either recovered (15 reports) or improved (12 reports) at the time of reporting. Seven patients improved slowly. Four patients were reported to have taken up to 9 months to recover and three had persisting mild increases in transaminases 6 to 12 months after stopping *A. annua*. The outcome was unknown for the remaining two patients. None of the reports indicated that the patients recommenced *A. annua* extract.

### Confounding Factors

#### Co-Suspect and Concomitant Medicines

In two reports, co-suspect medicines were indicated: turmeric and melatonin, respectively.

Fifteen reports listed concomitant medicines. Among the concomitants were several drugs that are known to be hepatotoxic, including atorvastatin (3 reports), diclofenac (1 report), cyproterone acetate (1 report), mesalazine (1 report), and paracetamol (3 reports). All but two of these concomitant medicines were continued with dose unchanged following the onset of hepatotoxicity, and the patient either recovered or improved while still taking these medicines. The patient taking mesalazine and one of the patients taking atorvastatin stopped these medicines but they were not considered suspect as the patients had been taking them for long periods. It is unknown whether or not improvement would have occurred if the concomitant medicines had been stopped as opposed to the *A. annua* extract. It is possible that hepatotoxicity was due to the combination of medicines.

#### Viral Serology

One patient had evidence of recent Epstein-Barr virus infection and another had chronic hepatitis B. Both were reported to have inactive disease.

#### Alcohol

Fifteen reports included information on alcohol consumption. In 13 reports, alcohol use was within recommended limits. One male patient who was described as a “heavy” drinker had not changed his pattern of intake during exposure to *A. annua*, after the onset of hepatic injury, or during recovery. One patient was described as having a “moderate-to-heavy” alcohol intake.

### Causality Assessment

Twelve of the 29 reports of hepatotoxicity were assessed (following consensus) as having a “probable” causal relationship with *A. annua* extract. Thirteen reports were assessed as having a “possible” causal relationship. One report was assessed as "unlikely” as the hepatitis did not occur until 3 months after stopping *A. annua* extract. Three reports were “unassessable.” In each of these reports, the possibility of onset of symptoms prior to use of *A. annua* extract could not be excluded, and no information was provided on investigation for other possible causes.

## Discussion

We present 29 case reports from the NZPhvC, of hepatic dysfunction or injury in patients taking *A. annua* herb extract. The hepatic events ranged from mild increases in hepatic enzymes to severe hepatitis, including one case of cirrhosis. Just over half of the patients in this case series presented with jaundice. Recovery or improvement on stopping *A. annua* extract was reported in 27 cases, although in some cases recovery took several months. Few reports included information that suggested alternative explanations for the hepatic events. Causality assessment supported a “probable” or “possible” causal relationship for 12 and 13 reports, respectively. While the proportion of “probable” reports is only 41%, this is usual for initial safety signals because of the limitations of examining data retrospectively. Most of the “possible” reports had missing data for causality assessment rather than equally likely alternative causes for hepatic disorders. The case reports represent a safety signal that requires further investigation which may strengthen or weaken the signal.

### Other Case Reports

*A. annua* has a long history of traditional use, yet there are very few published reports of hepatotoxicity. There are, however, numerous reasons why adverse reactions following use of herbal medicines may not be identified or reported. Typically, individuals who use herbal medicines do not seek professional advice if they experience adverse effects, and use of herbal medicines is often not disclosed to healthcare professionals (Barnes, 2003). The lack of adverse reaction reports for these substances should not be interpreted as evidence of “safety.”

The WHO Global Database of Individual Case Safety Reports, VigiBase^®^, contains two recent non-New Zealand reports of hepatobiliary disorders associated with *A. annua* (Uppsala Monitoring Centre, 2019a; Uppsala Monitoring Centre, 2019b). The first report was confounded by the use of several herbal preparations but the second concerned a male who took *A. annua* 1.25 g daily for six weeks. He developed cholestatic icterus eight weeks after initiation, which was reported as serious (caused or prolonged hospitalization). The reaction abated after stopping *A. annua* and the patient recovered.

We identified two published case reports of hepatotoxicity to herbal medicines containing artemisinin. In 2008, a 52-year-old man developed hepatitis after taking artemisinin 200 mg three times daily for one week (Centers for Disease Control and Prevention, 2009). Clinical investigation did not reveal any other cause for the hepatitis. He recovered two weeks after stopping artemisinin.

A 43-year-old woman developed arthralgia and jaundice five weeks after she began taking artemisinin 125 mg orally two-to-three times daily (Kumar, 2015). A thorough clinical investigation did not reveal any structural, viral, or autoimmune cause for the hepatitis, and paracetamol concentrations were undetectable. Discontinuation of artemisinin resulted in gradual clinical and biochemical improvement, and the woman remained asymptomatic with normal liver function one year later.

The databases of the FDA's Adverse Event Reporting System (AERS) and its Center for Food Safety and Applied Nutrition (CFSAN) Adverse Event Reporting System (CAERS) hold an additional eight reports of hepatotoxicity in patients using artemisinin as self-treatment (US Food and Drug Administration, 2017). An expert eview of these case reports and the two published reports (Centers for Disease Control and Prevention, 2009; Kumar, 2015) noted that artemisinin was used for a longer duration and at a greater dose than for malaria treatment (typically 3 days) and suggested that this may have contributed to hepatotoxicity.

All of the above reports were potentially or actually subject to confounding and do not represent the opinions of the WHO, Uppsala Monitoring Centre or US FDA. Nevertheless, taken together they do raise a hypothesis for hepatoxicity with *A. annua* or artemisinin products. At present it is not clear if they add to the evidence in our case series since the nature and content of the products used may be very variable, and constituents other than *A. annua* in the extract implicated in our case series could be responsible. Nevertheless the reports taken together suggest that there should be ongoing vigilance of *A. annua* and artemisinin products.

### Clinical Trial Data

Hepatotoxicity associated with the *A. annua* extract Arthrem^®^ was reported in a randomized, double blind, placebo-controlled pilot study. Of the 28 subjects randomized to Arthrem^®^, one of 14 participants receiving high-dose Arthrem^®^ (300 mg twice daily) developed hepatitis, considered possibly related to the study medicine by the investigator (Stebbings et al., 2016). Thirty-four participants continued into an open-label, six-month safety extension study of Arthrem 150 mg twice daily. One patient withdrew because of elevated serum hepatic enzymes, considered unrelated to the study medicine by the investigator (Hunt et al., 2016).

### Chemical Composition

Possible explanations for the hepatotoxic effects observed in this case series include involvement of toxic constituents other than artemisinin, production of a toxic artifact during the extraction process, and/or adulterated or contaminated raw materials entering the *A. annua* or grapeseed oil supply chains.

The products implicated in the case series presented contained an (SC-CO_2_) extract of *A. annua* in grapeseed oil. Grapeseed oil is an edible oil used widely in the food and pharmaceutical industries. Grapeseed oil typically contains polyunsaturated fatty acids, particularly linoleic acid, as well as other bioactive compounds, including phytosterols, flavonoids, and phenolic acids; the precise profile of constituents varies depending on the grape cultivar, growing conditions, and the precise extraction technique and conditions. The method used for extracting grapeseed oil from grapeseeds was not stated on the labels of the implicated products. SC-CO_2_ extraction has emerged as a technique for extracting oil from grape (and other) seeds as it presents several advantages when compared with standard methods, such as use of solvents, or mechanical pressing (Ben Mohamed et al., 2016). No evidence for grapeseed oil-related hepatotoxicity was found in the literature. The WHO Global Database, VigiBase^®^, contains 24 reports of suspected ADRs in the hepatobiliary SOC for *Vitis vinifera* (grape), but all of these reports relate to products containing red vine leaf extract, or grapeseed or skin extract (containing oligomeric proanthocyanidins), and not grapeseed oil.

In addition to artemisinin, *A. annua* extracts contain variable amounts of other constituents, including a series of arteannuins, artemisitine, artemisinic acid, flavonoids, including artemetin, and a volatile oil (Heinrich et al., 2018). The pharmacological activities of many of these compounds are not fully understood. The quantity of particular constituents in the raw herbal material is influenced by numerous factors, such as the growing location and conditions, and the time of harvest (Zhang et al., 2017). The concentration of artemisinin is highest in the leaves just before the plant flowers (DerMarderosian and Beutler, 2014). Further, the chemical composition of extracts prepared from *A. annua* leaves differs depending on the extraction method used (Iqbal et al., 2012).

Arthrem^®^ and Go-Arthri^®^ are prepared from *A. annua* by supercritical CO_2_ extraction of dried plant material (Promisia, 2019; GO Healthy, 2019). This method relies on the fact that carbon dioxide behaves as a liquid when under high pressure and is highly effective for extracting biomass (Heinrich et al., 2018). This method is not employed in China for *A. annua* extractions (personal communication) (Wang, 2019).

Arthrem^®^ capsules were obtained and analyzed in New Zealand and no adulterants were identified. Further analysis of product samples may provide insights into possible causes of the toxicity.

### Strengths and Limitations

The case reports were well documented due to the quality of reporting by health care professionals in New Zealand and their willingness to provide further information on request to assist with causality assessment. Whilst substantial efforts were made to collect missing information, for some reports non-exclusion of alternative causes, such as alcohol use and lack of description of the pattern of hepatic injury, limited causality assessment. Incomplete information is a well-known limitation for case series involving spontaneous adverse reaction reports.

The updated RUCAM method for causality assessment is considered the most appropriate method to apply when drug- or herb-induced liver injury is suspected. However, this was designed for prospective use and is problematic for assessing case reports retrospectively.^31^ Patients in this case series often improved on stopping the *A. annua* product so that testing beyond viral hepatitis studies and scanning was unnecessary for their clinical management. Also patients were not routinely tested for hepatitis E which is listed in the updated RUCAM method. While dates showed improvement in a reasonable time interval in most reports these were sometimes too imprecise for a RUCAM assessment. Nevertheless, in using the WHO-UMC causality assessment method we did familiarize ourselves with the RUCAM criteria to ensure that we considered relevant co-morbidities, the potential role of concomitant medicines, and times to onset or recovery which were unreasonable. On receiving follow up information the reports were reassessed by RS using the RUCAM method and five could be assigned to the “probable” category. The assignment to the “possible” rather than “probable” category in most cases was because detailed investigations were lacking rather than alternative causes being possible explanations.

In addition to causality assessment of the individual case reports we also looked for consistency and patterns across the case series. Our case series fulfills several of the Bradford Hill guidelines for causal inference (Shakir and Layton, 2002).

- Temporality: Onset of hepatotoxicity occurred within 3 to 4 months in most cases.
- Consistency: Internal consistency within the case series from various reporters and provinces in New Zealand and external consistency with other cases reported in the literature and in VigiBase for herbal products containing artemisinin or *A. annua* herb.
- Specificity: *A. annua* extract was usually the sole suspect medicine and hepatotoxicity the suspected adverse drug reaction in the reports. The pattern of liver injury was not consistent for all cases, but drug-induced liver injury takes various forms (Larson, 2018).
- Experimental evidence: One patient developed hepatotoxicity in the randomized controlled trial of Arthrem^®^ we have described and one in the extension study (Stebbings et al., 2016; Hunt et al., 2016).

### Population Exposure to *A. annua* Extract

This New Zealand case series emerged during a period when products containing *A. annua* were heavily promoted, achieving large sales volumes over a short time-period (Promisia Integrative Limited, 2016). This wide-spread exposure to *A. annua* extract may have facilitated the rapid detection of this safety signal.

## Conclusion

This case series has identified a signal for hepatotoxicity associated with products containing an extract of *A. annua* produced using a SC-CO_2_ extraction method, formulated with grapeseed oil (extraction method for grapeseed oil not stated on product label). The limitations to causality assessment of retrospective data and the uncertainty as to which constituent of the suspect product is implicated means that further investigation is required. This should include additional analysis of product samples, more rigorous experimental studies, and increased vigilance of *A. annua*-containing products.

## Data Availability Statement

The datasets analyzed in this manuscript are not publicly available. Requests to access the datasets that informed the study should be directed to ruth.savage@otago.ac.nz. 

## Ethics Statement

As the data was based on voluntary reports to the NZPhvC and no identifiable information is presented, informed consent was not obtained.

## Author Contributions

RS: Recognition of this case series as a suspected serious and unexpected adverse reaction while assessing spontaneous reports of adverse reactions. Assessment of each report. Liaison with the other authors. Wrote first, several and final drafts of paper. Participated in causality assessment with the other authors. Final responsibility for the article. GH: Addition of medicines regulatory expertise. Also assessed all of the reports. Major contribution to the writing and peer review of the paper. JB: Addition of expertise in herbal medicines aspects. Contribution to causality assessment. Major contribution to the writing and revision of paper. SK: Addition of regulatory expertise which contributed appropriate wording in the paper. Peer-review of final drafts of paper. MT: Overall responsibility for the conduct of the study, assessment of reports and contribution to drafting the paper.

## Conflict of Interest

The authors declare that the research was conducted in the absence of any commercial or financial relationships that could be construed as a potential conflict of interest.
